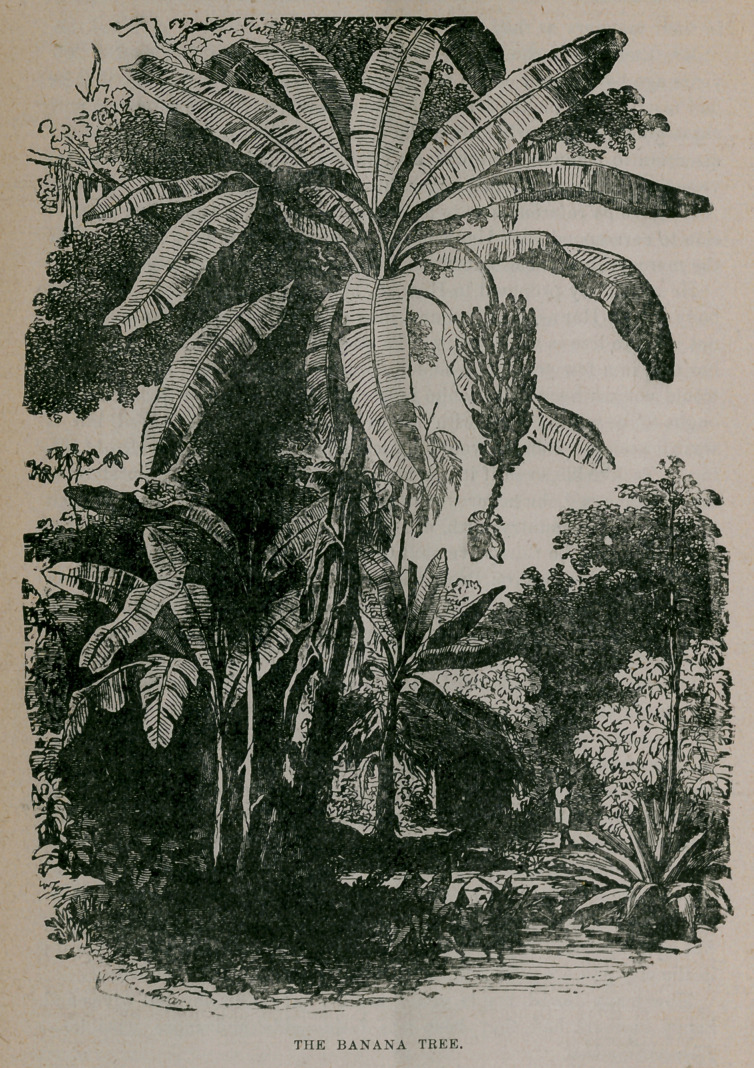# The Banana Tree

**Published:** 1888-05

**Authors:** 


THE BANANA TREE.

### THE BANANA TREE.

The banana is a variety of the plantain family, and is a native of the tropics. It is largely used as food, and cultivated for exportation. With the exception of two or three palms, it would not be easy to name in the whole vegetable kingdom any plant which is applied to a greater number of uses than the plantain.

The stem of the plantain, or banana, is from fifteen to twenty feet high, although there are varieties having a stem of only six feet. The leaves are very large, the blade being sometimes ten feet long and three feet broad, undivided, of a beautiful shining green. The plant is propogated by suckers, and a sucker attains maturity in about eight months or a year after being planted. The stem is cut down after fruiting, but the plantation does not require renewal for fifteen or twenty years. It has been cultivated successfully in hot-houses. Our illustration shows the banana in full growth, with stem of ripe fruit.

More than a hundred bananas often grow on a single stem, and so closely do they grow together, that tarantulas, the deadly insect of the tropics, are sometimes brought to the North concealed among them, and even small snakes have been found by the dealers when unpacking the fruit. The banana grows more in favor each year, and no place is too remote for its exportation. But to walk through the markets of a southern city, where bananas are for sale on every hand for almost nothing, and note the immense quantities in every stage of ripeness, it would seem as if they must decay on their stalks, so glutted is the market with this fruit alone.

## Figures and Tables

**Figure f1:**